# Application of Struvite-MAP Crystallization Reactor for Treating Cattle Manure Anaerobic Digested Slurry: Nitrogen and Phosphorus Recovery and Crystal Fertilizer Efficiency in Plant Trials

**DOI:** 10.3390/ijerph15071397

**Published:** 2018-07-03

**Authors:** Weijia Gong, Yan Li, Lina Luo, Xinsheng Luo, Xiaoxiang Cheng, Heng Liang

**Affiliations:** 1 School of Engineering, Northeast Agriculture University, 59 Mucai Street, Xiangfang District, Harbin 150030, China; liyanneau@163.com (Y.L.); luolina21333@163.com (L.L.); 2 State Key Laboratory of Urban Water Resource and Environment (SKLUWRE), Harbin Institute of Technology, 73 Huanghe Road, Nangang District, Harbin 150090, China; luoxinsheng2008@163.com (X.L.); cxx19890823@163.com (X.C.); hitliangheng@163.com (H.L.)

**Keywords:** cattle manure anaerobic digested slurry, MAP crystallization, phosphorus recovery, wastewater treatment, sustainability

## Abstract

Recycling and reusing the nutrient resources from anaerobic digested slurry is very promising for environmental pollution control and agriculture sustainable development. We focus here on nitrogen and phosphorus recycling in treating cattle manure anaerobic digested slurry by a magnesium ammonium phosphate (struvite-MAP) crystallization process and examine the impact of MAP precipitation on plant growth. The MAP crystallization process was studied by a combination of Design-Expert 8.0.6 software, mathematical modeling, and experiments. The influence of Mg/P, N/P and pH on nitrogen (N) and phosphorus (P) recovery was investigated. Then, the fertilizing efficiency of the MAP precipitate on the growth of three vegetables (water spinach (Swamp cabbage), amaranth and Brassica parachinensis) was also evaluated. The results showed that more than 89% of N and 99% of P could be recovered at pH = 10 with molar ratios of Mg/P = 1.6 and N/P = 1.2. Compared with the control pots and potassium chloridepots, the fresh weight, dry weight and average height of swamp cabbage in the MAP pots were obviously enhanced without burning effects. The results showed that MAP precipitation can promote the development of plants, which is promising for its use as a slow-release fertilizer for agricultural production.

## 1. Introduction

In recent years, biogas projects have been widely used to treat the manure and sewage of poultry and livestock. These projects achieve concentrated waste treatment and resource reuse, which is beneficial for environmental protection, energy saving and economic growth [1]. Due to the limitations of technology, management, and operational costs, biogas slurry is produced after anaerobic fermentation and this is a serious threat to the ecological environment and a major restriction to the development of biogas project as well [2,3]. However, high concentrations of nutrient elements such as nitrogen (N) and phosphorus (P) in the biogas sludge make the biogas slurry valuable for reuse. Phosphorus, a valuable resource which is non-renewable and has limited availability in nature, also has the potential to cause eutrophication or blue green algal blooms in receiving waters [4,5]. Therefore, an appropriate method for recycling nitrogen and phosphorus nutrients from biogas slurry to agricultural production is necessary [2,6,7].

Magnesium ammonium phosphate (MgNH_4_PO_4_·6H_2_O, MAP) crystallization is regarded as an effective method for P and N recycling from the slurry, and MAP precipitation can be used as a potential slow-release fertilizer for crop growth [8]. If MAP precipitation could be directly used for agricultural production, this would reduce the need for mined phosphorus and synthetic nitrogen resources. The basic principle of MAP crystallization is that magnesium sources react with PO_4_^3−^ and NH_4_^+^, which simultaneously remove nitrogen and phosphorus from wastewater by MAP crystallization [9]. The main chemical reaction is as follows in Equation (1):

Mg^2+^ + NH_4_^+^ + PO_4_^3−^ + 6H_2_O → MgNH_4_PO_4_·6H_2_O (1)

In recent years, the treatment of wastewater with the MAP crystallization process has become a topic of great interest for many researchers and some progress has been made. For example, Qian et al. [10] studied the MAP crystal collecting reactor from anaerobic digested piggery wastewater and found a high P recovery rate of 82% under aeration condition. Bao et al. [11] investigated phosphorus recovery from swine wastewater, and analyzed different types of precipitated minerals under different pH conditions. They found that very pure struvite crystal could be obtained at pH 8.0–9.0. Yilmazel et al. [12] recovered nutrients from anaerobic digested slurry. The Mg:N:P molar ratio of 1:1:1 in the liquid phase resulted in an average NH_4_-N removal efficiency of 86.4%, which increased to 97.4% by adjusting the Mg:N:P ratio to 1.5:1:1. Based on the above research, the current study of MAP crystallization sewage treatment focused on basic reaction indicators, including the molar ratio of reactive ion and pH, etc. [13,14,15,16]. Few studies have explored the effects of MAP precipitation on plant growth. In this paper, a MAP crystallization reactor was used to study the recovery of nitrogen and phosphorus in treating cattle manure anaerobic digested slurry. Moreover, we took the recovery rate of P and N as the performance indexes, and mole ratio Mg/P, N/P and pH as the influence factors to carry out three-factor and three-level orthogonal experiments [7,17,18]. Then, the fertilizer efficiency of the MAP precipitate on plant growth was evaluated by pot trial tests.

## 2. Material and Methods

### 2.1. Material

The cattle manure anaerobic digested slurry was taken from a cow dung anaerobic digestion tank in the HeiLongJiang ShuangCheng biogas fermentation project. After centrifuging at 10,000 rpm for 30 min, all samples were stored below 4 °C for further experiments. The NaOH, NH_4_Cl, Na_2_HPO_4_·12H_2_O, KCl and MgCl_2_·6H_2_O used in the experiments were purchased from Tianjin Damao Chemical Reagent Co., Ltd. (Tianjin, China). All the chemicals and reagents used were analytical grade.

### 2.2. Influent Characteristics

The concentration of ammonium-N (NH_4_^+^-N) and phosphate-P (PO_4_^3−^) in the cattle manure anaerobic digested slurry arranged from 100 to 700 mg/L and 10 to 60 mg/L, respectively. The stock solution was prepared by dissolving certain amount of NH_4_Cl and Na_2_HPO_4_·12H_2_O in cattle manure anaerobic digested slurry. The initial concentration of NH_4_^+^-N and PO_4_^3−^ in the solution was 450 mg/L and 60 mg/L, respectively [8,19]. MgCl_2_ and Na_2_HPO_4_ were used as magnesium and phosphorus source. The solution pH was adjusted by adding 5 mol/L NaOH solution.

### 2.3. Design of Reaction Equipment

**Figure 1:**
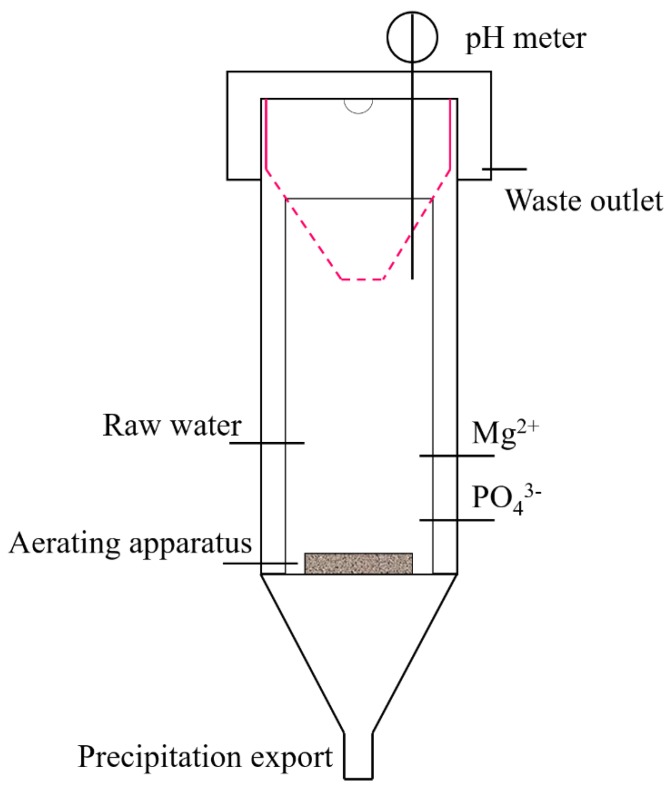
Schematic diagram of the magnesium ammonium phosphate (MAP) reaction equipment.

The MAP crystallization reactor was designed in conical shape and made of polymethyl methacrylate material with an effective volume of 6.69 L as shown in Figure 1. The reaction was divided into the inner and the outer cylinder. The inner cylinder with a stainless-steel screen at the top is the main reaction site, which was used for collecting some crystals. The outer cylinder was the side reaction site, and the bottom of the cylinder was provided with an inlet for the feed liquid, an inlet for chemicals (NH_4_Cl, Na_2_HPO_4_·12H_2_O and MgCl_2_) and an inlet for the lye (Figure 1). The bottom was the sedimentation area used for precipitation and collection of crystals. The experimental process was carried out in continuous water. The simulated wastewater, magnesium source and phosphorus source were pumped into the device by a peristaltic pump. The Mg/P and N/P of the mixture was controlled as a certain value. The reaction was mixed by aeration (the aeration head is placed at the bottom of the reactor). The pH of the solution was detected by pH meter and controlled by NaOH.

In order to explore the optimal reaction conditions of the MAP crystallization process, orthogonal tests based on three factors and three levels were designed and carried out to systematically study the influence of the mole ratios, Mg/P, N/P and pH on the recovery rate of P and N as the performance indexes. It is known from previous experiments, the influent flow rate of the crystallization reactor had less effect on the MAP crystallization reaction, so it was not considered, and all of the influent flow rates of the experiments were fixed at 1.9 L/h. The experimental factors and levels are shown in Table 1.

### 2.4. Design of Pot Trial Tests

To explore the effect of adding MAP to the plants, the MAP precipitated under the optimized conditions of 1.2, 1.6 and 9 for N/P, Mg/P and pH, respectively, was collected as vegetable fertilizer. Then, three species of quick-growth vegetables including swamp cabbage, amaranth and Brassica parachinensis Bariley were selected for the pot trial tests at room temperature. The soil samples were taken from the west side lawn of the School of Engineering of Northeast Agricultural University and sieved with a maximum aperture size of 2.36 mm to remove weeds and gravel. Each pot contained 1.4 kg of soil according to the size of the plastic flowerpot (top diameter: 15 cm, height: 13.5 cm and bottom diameter: 11.7 cm). The parameters of the raw soil were: pH = 7.7, ammonia nitrogen content = 0.44 mgN/g, and moisture content = 14.28%. In this study, the effect of adding MAP to the plants was compared with a single chemical fertilizer treatment, and each of the plant growth experiments were conducted in three different conditions: (1) raw soil only as control; (2) raw soil with addition of KCl; and (3) raw soil with addition of KCl and MAP precipitate. It was suggested that the optimum dosage of fertilizer in soil for Chinese vegetable growth was hydrolyzed nitrogen >70 mg/kg, exchangeable potassium >100–150 mg/kg and instant phosphorus >60–80 mg/kg. If the lower limits were applied, the required dosage of KCl and the MAP precipitate for each pot (1.4 kg soil) were 267 mg and 1.72 g, respectively. To investigate the influence of MAP dosage on plant growth, swamp cabbage (water spinach) was planted in four pots with different amounts of MAP added one time (1×), two times (2×), three times (3×) and four times (4×). The schematic diagram of the pot trial tests (greenhouse experiment) is shown in Figure S1 (in Supplementary Materials).

The vegetable cultivation proceeded as follows: (1) each pot was filled with 1.4 kg of sieved raw soil; (2) top layer of 2 cm was removed and evenly mixed with the pre-weighed chemicals as required in each test; (3) 100 mL of water was added into each pot and the vegetable seeds were laid evenly on the exposed surface of the soil; (4) the seeds were covered with the removed soil; (5) another 100 mL of water was added into each pot, the pots were placed outdoors and irrigated as required.

### 2.5. Analyses Methods

The pH of water samples and soil samples were measured by a pH meter (PHSJ-3F, Shanghai, China). Ammonium nitrogen (ammonium-N) was measured by Kjeldahl Apparatus (Kjeltec 2300). Orthophosphate (phosphate-P) was determined according to the Ammonium Molybdate Spectrophotometric Method. Moisture content was determined by Gravimetric Method [20].

For the vegetative samples, height was measured with a metric ruler. After harvest, the vegetative samples were measured by analytical balance, and then weighed before and after drying at 60 ± 5 °C.

## 3. Results and Discussion

### 3.1. Analyses of MAP Orthogonal Tests

The experimental data w analyzed by Design-Expert 8.0.6. The results are shown in Table 2.

### 3.2. Effects of Various Factors on the Recovery of Nitrogen and Phosphorus

The processed data was used to analyze the effects of various factors on the recovery of nitrogen (N) and phosphorus (P). The results are shown in Table 3.

#### 3.2.1. Effect of pH on the Reaction

**Figure 2:**
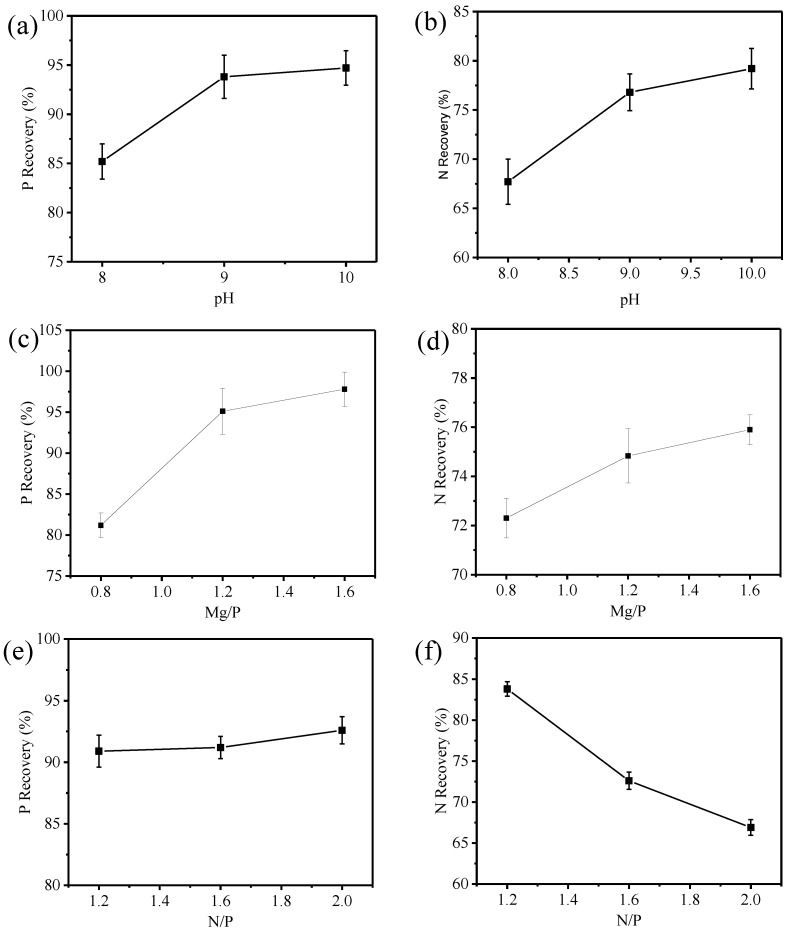
Effect of pH (**a**,**b**), Mg/P (**c**,**d**) and N/P (**e**,**f**) on the recovery of P and N, respectively.

It can be observed from Table 3 that pH has a significant influence on the recovery rate of N and P. The effect of pH on N and P recovery rate is shown in Figure 2.

pH is a critical factor for MAP crystallization, which determines the equilibrium concentration and type of the MAP formation ion. As shown in Figure 2a,b, the recovery rates of N and P rapidly increased with the increase in the pH ranging from 8 to 9. When the pH value further increased to 10, the recovery rate of P remained stable with approximately 95% (Figure 2a); whereas the recovery rate of N slowly increased to 78% when the pH value was higher than 9.0 (Figure 2b). This difference can be explained by the following reason: when pH is above 9, NH_4_^+^ can deprotonate into NH_3_ and this results in NH_3_ vaporization that inhibits the MAP crystallization process, thus increasing the recovery rate of N. The above results indicated that there was an optimal pH value for the recovery rate of N and P during the process of MAP reaction. In previous studies, Booker et al. [21] found the optimum pH for struvite formation in wastewater treatment plants was 9.2, and Liao et al. [22] indicated that the optimum pH for struvite formation in synthetic wastewater treatment was 10.

#### 3.2.2. Effect of Mg/P on the Reaction

As shown in Table 3, the effect of Mg/P on the recovery of P was significant, but its effect on the recovery of N was less. It is shown in Figure 2c,d that the recovery rate of P increased rapidly from 80.8% to 95.5% when Mg/P increased from 0.8 to 1.2. However, the recovery rate only increased 2% when the value of Mg/P increased from 1.2 to 1.6. As shown in Figure 2d, Mg/P had less effect on the recovery rate of N. The recovery rate of N only increased from 72.13% to 75.85% when the value of Mg/P increased from 0.8 to 1.6. Liu et al. [13] reported that the optimum Mg/P ratio for synthetic wastewater treatment was 1.3–1.5:1 with P removal rate of 89–92%. The above analysis suggests that the recovery of N and P from the anaerobic digested slurry can be promoted by increasing the molar ratio of Mg^2+^ and PO_4_^3−^ in the solution, but an excessive amount of magnesium results in drug waste and serious secondary pollution [23,24]. Thus, to ensure the recovery rate of N and P, Mg/P should be controlled within a reasonable range.

#### 3.2.3. Effect of N/P on the Reaction

N/P is also an important factor in the reaction. From Table 3, it could be seen that N/P has an obvious influence on N recovery rate, whereas P recovery rate is less affected. The influence of N/P on the recovery rate of N and P is shown in Figure 2e,f. As shown in Figure 2e, when N/P ranged from 1 to 1.2, it has less influence on the recovery rate of P. However, the concentrate of NH_4_^+^ increases when N/P decreases, which is not beneficial for the P recovery. As shown in Figure 2f, the recovery rate of N rapidly increases from 67.2% to 83.3% when N/P decreases from 2 to 1.2, which means that higher PO_4_^3−^ concentration is propitious for the recovery of N. Considering the recovery of P and the residual concentration of PO_4_^3−^, it was necessary to ensure that the concentration of NH_4_^+^ in the solution was excessive in comparison to the concentration of PO_4_^3−^ [16,25].

### 3.3. The Model Relationship between Response Value and Various Factors

As listed in Table 3, the main factors that affected the recovery of P and N were pH, Mg/P and N/P. In Design-Expert 8.0.6, two main factors were selected to establish the relationship model, and the results are shown in Formula (2) and (3). The variance analysis and simulation results of the model are shown in Table 4 and Figure S2 (in Supplementary Materials).

Recovery rate of P = 91.27 − 6.10A + 2.63A^2^ − 10.52B + 4.27B^2^ (2)

Recovery rate of N = 74.32 − 6.82A + 2.08A^2^ + 8.95C − 1.80C^2^ (3)

In the analysis of variance, the F value is an important index. With a larger F value and smaller *p* value, the analysis results would be considered more reliable. For example, if the value of *p* equals 0.01, the probability of the correct conclusion is 99%. In the analysis of Design-Expert 8.0.6, if the *p* value is less than 0.05, the model would be considered to be effective. Studentized residual is used to judge whether the data is an anomaly relative to the regression curve. If the value of residual is larger, the data point would be further from the fitting curve, which means the fitting effect is worse.

It can be seen from Table 4, the *p* value of all the models based on the recovery of P and N are less than 0.05, and the nine points in Figure S2 (in Supplementary Materials) are basically distributed on a straight line, which indicates that the models are well correlated with the experimental data.

### 3.4. Optimization of Experimental Scheme

According to the above analysis of the experimental results and model fitting, the experimental parameters were further optimized to explore the optimal scheme of various factors and levels for the highest recovery rate of N and P by Design-expert 8.0.6. “0 < recovery rate of N and P < 100%” was typed into the software to obtain the value of various factors by the software analysis, which could then be tested by the experiments.

The ratios of the optimized scheme are shown in Table 5. The predicted recovery rate of N and P are 89.6% and 99.6%, respectively. It was known from the single factor experiments that the recovery rate of N and P were 89.03% and 99.76%, respectively (precipitation condition: Mg/P = 1.6, N/P = 1.2 and pH = 10), which was consistent with the predicted theoretical conclusion in the error range.

### 3.5. XRD Analysis of Sedimentation

**Figure 3:**
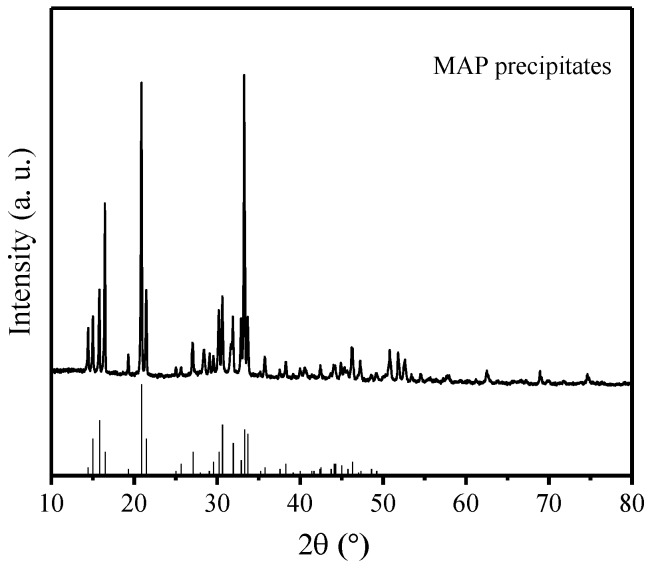
XRD patterns of MAP precipitates. The vertical lines in the figure denote the standard XRD patterns.

The crystalline phases in the precipitation were analyzed with X-ray diffraction. Figure 3 shows the XRD pattern of the precipitation under the condition of Mg/P = 1.6, N/P = 1.2 and pH = 10. The diffraction peaks were consistent with the values of the standard card. No other crystalline phase was shown in the figure, indicating the high purity of the MAP precipitates.

### 3.6. The Results of Pot Trial Tests

**Figure 4:**
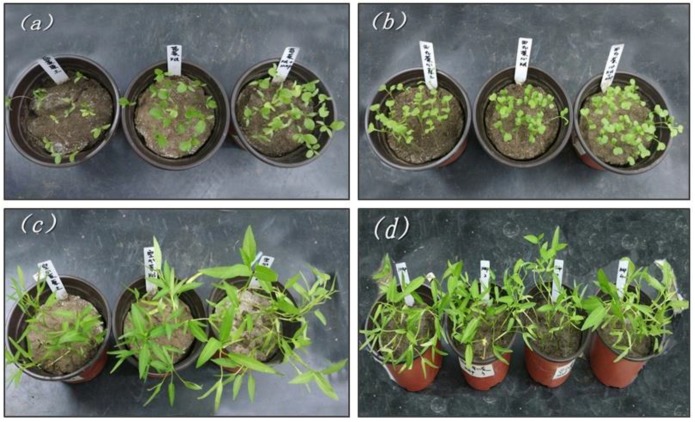
Photos of three vegetables at different cultivation condition after 35 days growth. (left, control pots; middle, KCl pots; right, MAP pots) (**a**) Amaranth. (**b**) Brassica parachinensis Bariley. (**c**) Swamp cabbage. (**d**) Gradient test of swamp cabbage. (From left to right: 1×, 2×, 3×, and 4× MAP).

**Figure 5:**
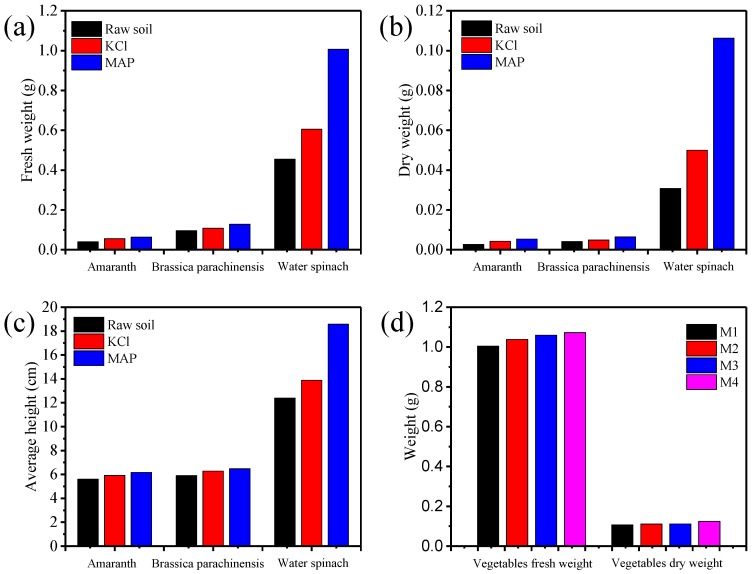
Fresh weight (**a**), dry weight (**b**) and average height (**c**) of vegetables after 35 days growth. Fresh weight and dry weight of four individual swamp cabbages with different MAP dose (**d**).

After seeding, the vegetables sprouted for about 5 days. It was observed that the seedlings in the three pots looked very similar. Figure 4 shows the photos of three vegetables at different cultivation condition after 35 days growth. As illustrated in Figure 4, the vegetables in the MAP pots grew faster than the other two. All vegetables were harvested on the 35th day after sowing. Before the harvest, the vegetables in the pots were sprayed with water to wash the dust off. After drying in ambient conditions, the height of each plant was measured with a metric ruler and the average height was calculated (Figure 5c). After measuring the height, the cut vegetable samples were weighed before and after drying at 60 ± 5 °C. Their fresh weight and dry weight were recorded and compared as shown in Figure 5a,b.

As can be observed from Figure 5a,b, both the fresh weight and the dry weight of the vegetables grown in the MAP pots were heavier than the vegetables growing in the control pots and KCl pots, which indicates that the MAP precipitation effectively stimulates the growth of plants. The fresh weight and dry weight of amaranth and Brassica parachinensis Bariley in the MAP pots, control pots and KCl pots were contiguous. However, the fresh weight and dry weight of swamp cabbage in the MAP pots were much heavier than those in the control and KCl pots. The fresh weight of swamp cabbage in the MAP pots was 57% and 43% heavier than those in the control pots and KCl pots, respectively. The dry weight of swamp cabbage in the MAP pots was 71% and 53% heavier than those in the control pots and KCl pots, respectively. This discrepancy might be caused by the different growth patterns of plant species and different nutritional requirements [15,24]. There was no doubt that the MAP precipitation could be used as a slow-release fertilizer to release nutrients slowly and provide good nutrition for plants in the process of plant growth, especially for swamp cabbage. Likewise, the average height of the vegetables after harvest in the MAP pots was higher than the other two [14].

In another set of experiments, swamp cabbage was cultivated in four pots with different amounts of MAP added, that is 1×, 2×, 3× and 4× MAP (denoted by M1, M2, M3 and M4, respectively). The growth condition is shown in Figure 4d. It was observed that the vegetables in all pots grew quite well, and there were no burning effects on the growth of swamp cabbage, which demonstrated that the excessive application of MAP in a certain range was not harmful to the plants. The vegetables were harvested after 35 days. The average fresh weight and dry weight of the swamp cabbage in each pot were compared as shown in Figure 5d. The weight of the vegetables increased with the addition of more MAP, which illustrated that the addition of more MAP could stimulate the growth of the vegetable [3,14]. However, the effect of the stimulation was not obvious.

According to the above analysis, MAP precipitation could effectively promote the growth of plants (especially for swamp cabbage), and it is very promising for agricultural production as a slow-release fertilizer.

## 4. Conclusions

This work demonstrated the recycling of nitrogen and phosphorus from cattle manure anaerobic digested slurry by the MAP crystallization process. The following conclusions can be drawn:

(1) For the MAP crystallizer, the main factors influencing phosphorus recovery were pH and Mg/P, and the main influencing factors for nitrogen recovery were pH and N/P.

(2) Through analysis of the experimental results and model fitting by Design-expert 8.0.6, the experimental parameters could be optimized to explore the optimal scheme of various factors and levels for the highest recovery rate of N and P.

(3) More than 89% of N and 99% of P could be recovered at pH = 10, with the molar ratio of Mg/P = 1.6 and N/P = 1.2.

(4) Vegetables growing in pots with MAP precipitation as a slow-release fertilizer had advantages over the vegetables growing in the control pots and KCl pots with respect to fresh weight, dry weight and average height. The results indicated that MAP precipitation could be used directly as an efficient slow-release fertilizer.

## Figures and Tables

**Table 1:** Factors and levels in the orthogonal tests.

Levels	Factors		
	pH (A)	Mg/P (B)	N/P (C)
1	8	0.8	1.2
2	9	1.2	1.6
3	10	1.6	2

**Table 2:** Results analysis of the orthogonal tests.

Sequence	Factors			Recovery Rate of P (%)	Recovery Rate of N (%)
	pH	Mg/P	N/P		
1	8	0.8	1.2	72.5	73.5
2	8	1.2	1.6	88.8	66.8
3	8	1.6	2	94.2	62.2
4	9	0.8	1.6	84.1	72.7
5	9	1.2	2	99.1	69.1
6	9	1.6	1.2	98.5	87.4
7	10	0.8	2	85.7	70.2
8	10	1.2	1.2	98.7	89.0
9	10	1.6	1.6	99.8	78.0

**Table 3:** Variance analysis of the experimental results.

Variance Source		A	B	C
Recovery rate of P	Quadratic sum	168.7	503.9	15.3
	Degree of freedom	2	2	2
	Mean square	84.4	251.9	7.7
Recovery rate of N	Quadratic sum	219.9	22.9	403.4
	Degree of freedom	2	2	2
	Mean square	109.9	11.5	201.7

**Table 4:** Variance analysis of the model.

Variance Source		Quadratic Sum	Degree of Freedom	Mean Square	F Value	*p* Value (>F)
Recovery rate of P	pH	168.69	2	84.35	31.32	0.0309
	Mg/P	503.86	2	251.93	93.55	0.0106
Model		687.88	6	114.65	42.57	0.0231
Recovery rate of N	pH	219.97	2	109.99	79.86	0.0124
	N/P	403.39	2	201.7	146.45	0.0068
Model		646.27	6	107.71	78.21	0.0127

**Table 5:** The ratios of the optimized scheme.

pH	Mg/P	N/P	Recovery Rate of P (%)	Recovery Rate of N (%)	Reliability (%)
10	1.6	1.2	99.6	89.6	99.6

## Supplementary Materials

The following are available online at http://www.mdpi.com/1660-4601/15/7/1397/s1, Figure S1: Schematic diagram of the pot trial tests, Figure S2, Residual distribution of the recovery rate of P (a) and N (b).
