# Supplementary material for: Application of Struvite-MAP Crystallization Reactor for Treating Cattle Manure Anaerobic Digested Slurry: Nitrogen and Phosphorus Recovery and Crystal Fertilizer Efficiency in Plant Trials
Source: Int J Environ Res Public Health. 2018 Jul 3;15(7):1397. doi: 10.3390/ijerph15071397 (PMC6069237; doi:10.3390/ijerph15071397)

Supplementary data:

Application of Struvite-MAP Crystallization Reactor for Treating Cattle Manure Anaerobic Digested Slurry: Nitrogen and Phosphorus Recovery and Crystal Fertilizer Efficiency in Plant Trials

Weijia Gong [1,*], Yan Li [1], Lina Luo [1], Xinsheng Luo [2], Xiaoxiang Cheng [2] and Heng Liang [2]

[1] School of Engineering, Northeast Agriculture University, 59 Mucai Street, Xiangfang District, Harbin 150030, China; liyanneau@163.com (Y.L.); luolina21333@163.com (L.L.)

[2] State Key Laboratory of Urban Water Resource and Environment (SKLUWRE), Harbin Institute of Technology, 73 Huanghe Road, Nangang District, Harbin 150090, China; luoxinsheng2008@163.com (X.L.); cxx19890823@163.com (X.C.); hitliangheng@163.com (H.L.)

* Correspondence: gongweijia@126.com; Tel.: +86-451-5519-1760

Fig. S1 Schematic diagram of the pot trial tests

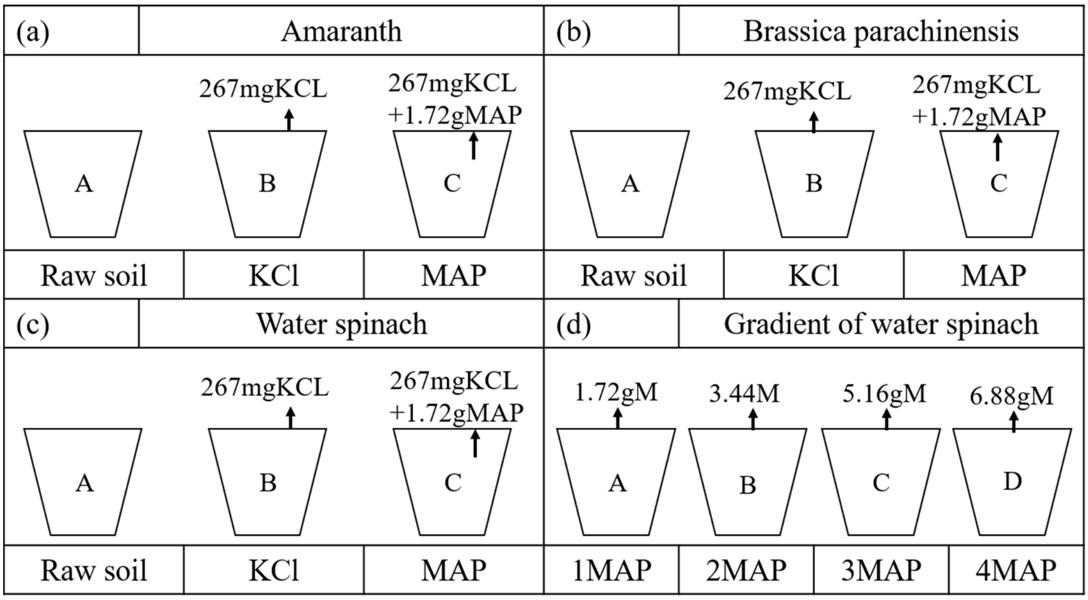

Fig. S2 Residual distribution of the recovery rate of P (a) and N (b)

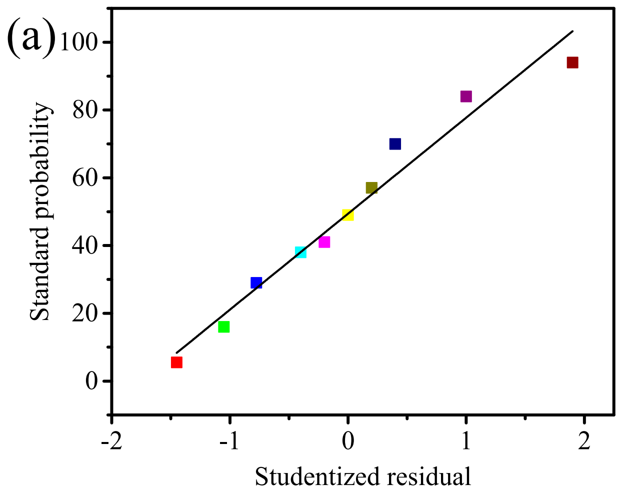

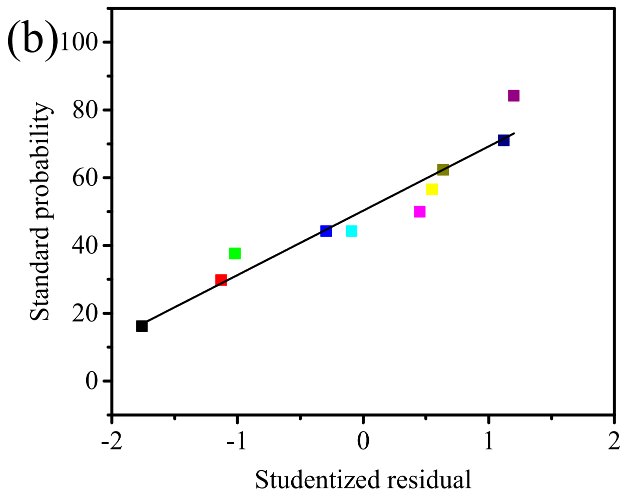

Supplement: Supplementary file 1 [file ijerph-15-01397-s001.pdf]
